# Multiplex Analysis of Serum Cytokines in Humans with Hantavirus Pulmonary Syndrome

**DOI:** 10.3389/fimmu.2015.00432

**Published:** 2015-08-31

**Authors:** Sergey P. Morzunov, Svetlana F. Khaiboullina, Stephen St. Jeor, Albert A. Rizvanov, Vincent C. Lombardi

**Affiliations:** ^1^Department of Pathology, School of Medicine, University of Nevada, Reno, NV, USA; ^2^Institute of Fundamental Medicine and Biology, Kazan Federal University, Kazan, Russia; ^3^Whittemore Peterson Institute, Reno, NV, USA; ^4^Department of Microbiology and Immunology, University of Nevada, Reno, NV, USA; ^5^Department of Biochemistry, School of Medicine, University of Nevada, Reno, NV, USA

**Keywords:** hantavirus pulmonary syndrome, serum, cytokines, chemokines, growth factors, immune response, hantaviruses

## Abstract

Hantavirus pulmonary syndrome (HPS) is an acute zoonotic disease transmitted primarily through inhalation of virus-contaminated aerosols. Hantavirus infection of endothelial cells leads to increased vascular permeability without a visible cytopathic effect. For this reason, it has been suggested that the pathogenesis of HPS is indirect with immune responses, such as cytokine production, playing a dominant role. In order to investigate their potential contribution to HPS pathogenesis, we analyzed the serum of hantavirus-infected subjects and healthy controls for 68 different cytokines, chemokines, angiogenic, and growth factors. Our analysis identified differential expression of cytokines that promote tissue migration of mononuclear cells including T lymphocytes, natural killer cells, and dendritic cells. Additionally, we observed a significant upregulation of cytokines known to regulate leukocyte migration and subsequent repair of lung tissue, as well as cytokines known to increase endothelial monolayer permeability and facilitate leukocyte transendothelial migration. Conversely, we observed a downregulation of cytokines associated with platelet numbers and function, consistent with the thrombocytopenia observed in subjects with HPS. This study corroborates clinical findings and extends our current knowledge regarding immunological and laboratory findings in subjects with HPS.

## Introduction

Hantavirus pulmonary syndrome (HPS) is a severe life threatening disease caused by members of the genus *Hantavirus*. In the United States, these members include Sin Nombre virus, Bayou virus, Black Creek Canal virus, and New York virus, while South American members include Andes virus and Laguna Negra virus (1–5). Although HPS was first diagnosed as a clinical entity in 1993 in response to the four corners outbreak (6), retrospective studies have identified hantavirus-associated fatalities as early as 1978 (7). HPS cases have been reported in 34 states with the majority occurring in the Southwestern states; however, several have been reported in the Northwestern and Midwestern states. Through April 2014, the Center for Disease Control and Prevention has confirmed 639 total cases of HPS in the U.S., with the majority occurring in New Mexico (94 cases), Colorado (81 cases), and Arizona (72 cases) (8). Although the prevalence of HPS is low in the U.S., 36% of all reported HPS cases have resulted in death, underscoring the potential impact to public health.

Clinically, HPS manifests with fatigue, fever, muscle pain, headache, dizziness, nausea, and vomiting (9). Soon after onset, individuals present with bilateral diffuse interstitial edema resembling acute respiratory distress syndrome (10). Rapidly progressing pulmonary edema, myocardial depression, and hypovolemia are the leading cause of death (11). There is no specific treatment for HPS; therefore, medical care is mainly supportive with early diagnosis resulting in more successful outcomes.

Hantaviruses do not produce a visible cytopathic effect; consequently, it is believed that cytokines produced by infected cells either directly or indirectly lead to a compromised endothelial monolayer, which in turn, leads to vascular leakage. Indeed, increased numbers of cytokine-producing cells have been observed in lung and spleen tissue of HPS cases (12). We as well as others have demonstrated that endothelial cells produce the chemokines, CCL5 and CXCL10, when infected with Sin Nombre virus (13, 14). These cytokines are strong chemoattractants for mononuclear leukocytes including monocytes, lymphocytes, and natural killer (NK) cells (15, 16). Expression of these chemokines may explain the postmortem observation of monocytic interstitial pneumonia in fatal HPS cases; however, it remains to be determined whether these chemokines are expressed during active HPS. In contrast to CCL5 and CXCL10 and atypical of most viral infections, *in vitro* culture studies show that only a slight upregulation of type I interferon (IFN) is observed when endothelial cells are infected with hantaviruses. These data are also consistent with clinical observations that suggest that a robust IFN-α response is not characteristic of hantavirus infection (17, 18).

Although limited data exist regarding cytokine expression in subjects with HPS, a study by Borges et al. evaluated the concentrations of 11 serum analytes by ELISA. A cytokine profile was reported that defined the differential expression of a selected number of Th1 and Th2 cytokines (19). Specifically, they observed significantly elevated levels of IL-6, IFN-γ, sIL-2R, TNF-α, and decreased IL-10 when compared to controls, suggesting that activation of Th1 and Th2-type immune responses are involved. While ELISA is commonly used for such studies, it has limitations such as the necessity of a large sample volume and this issue is compounded when one wishes to analyze multiple analytes. High-throughput multiplex analysis by Luminex xMAP technology allows the simultaneous detection and quantitation of many analytes and uses a small amount of serum or plasma. In the present study, we utilized Luminex xMAP technology to conduct a comprehensive evaluation of 68 different cytokines, chemokines, angiogenic, and growth factors (hereafter referred to collectively as cytokines) in subjects with HPS, including 38 cytokines previously not investigated in association with this disease. Changes in 40 cytokines were detected in the serum of subjects with HPS when compared to healthy controls; 25 cytokines were significantly upregulated while15 were downregulated. A subset of these cytokines known to influence the migration of mononuclear effectors was upregulated, as were cytokines known to play a role in lung microbial defense and tissue repair. Another subset of cytokines associated with thrombocyte counts and function was downregulated. This study corroborates clinical findings and extends our current knowledge by providing a more comprehensive basis for the immune responses and morphology observed in laboratory and histological findings in subjects with HPS.

## Materials and Methods

### Subjects

Twelve clinical diagnostic serum specimens collected from 2008 to 2012 by the Nevada State Health Laboratory (NSHL) and with a confirmed diagnosis of HPS were utilized in this study. The NSHL serves as a regional reference laboratory and routinely screens subjects suspected of having HPS, by the presence of anti-hantavirus antibodies. These deidentified diagnostic specimens were deemed to be exempt from IRB approval by the University of Nevada (UNR), Research Integrity Office (Reference #616225-1) as meeting the exemption criteria defined by the Department of Health and Human Services under Human Subject Research Code 45 CFR 46.102(f). Information of each HPS case was limited to diagnosis, gender, and antibody titer range. Forty-two serum samples from healthy individuals collected under informed consent were used as controls (Human subjects protocol # B12-031). Control subjects were chosen to be consistent with published demographics of typical HPS cases regarding age and gender (male to female ratio of 54–46%, respectively, and mean age of 39.4 years) (20).

### HPS screening

Serum anti-hantavirus antibody titers were evaluated by ELISA, according to the methods described by Feldmann et al. (21). Serum dilutions (1:100–1:6400) were tested for the presence of anti-hantavirus IgG and IgM using recombinant nucleocapsid protein supplied by the United States Centers for Disease Control and Prevention (CDC, Atlanta, GA, USA). Subjects with antibody titers greater than twofold above that of negative controls were considered positive.

### Multiplex analysis

The levels of serum cytokines were analyzed using Bio-Plex (Bio-Rad, Hercules, CA, USA) multiplex magnetic bead-based antibody detection kits following the manufacturer’s instructions. The Bio-Plex Pro Human Chemokine Panel (40-Plex); Bio-Plex Pro Human Th17 Cytokine Panel; Bio-Plex Pro Human Cytokine 27-plex Panel; and Bio-Plex Human Cytokine 21-plex Panel were used for analysis of a total of 68 analytes. Fifty microliters of serum from each respective case and control was analyzed using a Luminex 200 analyzer with MasterPlex CT control software and MasterPlex QT analysis software (MiraiBio, San Bruno, CA, USA). Standard curves for each analyte were generated using standards provided by manufacturer. Serum samples from HPS cases were heat inactivated and tested for the presence of infectious virus prior to Luminex analysis. The effect of heat inactivation on cytokine stability was evaluated and those that could not be normalized were excluded from analysis.

### Statistical analysis

Mann–Whitney non-parametric analysis was utilized to identify differences in medians between HPS cases and controls. In addition, we performed classification analysis using the tree-based ensemble machine learning algorithm Random Forest (RF) (22). For this analysis, 500 random trees were built using six predictors for each node, and auto-bootstrap out-of-bag sampling was used for testing the model as previously described (23).

## Results

### Anti-hantavirus titer in HPS serum

Twelve serum samples from subjects suspected of having hantavirus infection were tested for the presence of anti-hantavirus IgG and IgM antibodies. Antibody titers twofold greater than those of the control samples were considered diagnostic for hantavirus infection (Table 1). Previous reports suggest that anti-hantavirus IgM and IgG change with disease progression (24, 25). As reported by MacNeil and coworkers, early stage HPS is characterized by high IgM titers that peak within 11–14 days after onset whereas cases with early stage HPS often have no SNV-specific IgG titer (24). In contrast to IgM titers, median IgG titers typically displayed an increasing trend for a longer interval after the onset of disease. In light of the deidentified nature of our HPS cases, we used antibody titers to assess the stage of their illness. Six of our cases had high serum titer of IgM while IgG levels were low or undetectable, indicative of early stage disease. For the remaining six cases, high serum titers were observed for both IgG and IgM, consistent with late onset HPS.

**Table 1:** **Antibody titer in serum from HPS cases**.

Subject	IgM titer	IgG titer	Stage
1	>6400	<400	Early
2	>6400	<400	Early
3	>6400	<400	Early
4	>400	Negative	Early
5	>400	Negative	Early
6	>400	Negative	Early
7	<6400	>6400	Late
8	<6400	>6400	Late
9	<6400	>6400	Late
10	<6400	>6400	Late
11	<6400	>6400	Late
12	<6400	>6400	Late

### Differential expression of serum cytokine in HPS cases

A total of 68 serum cytokines were measured for HPS cases and controls (Tables 2–4). To the best of our knowledge, 38 of these cytokines were previously uninvestigated in the context of HPS (indicated by an asterisk in Tables 2–4). A significant increase in the serum levels of 25 of 68 (36.7%) cytokines were observed for the HPS cases when compared to healthy controls (Table 2). The greatest difference was observed for IL-6, CXCL10, CX3CL1, MIF, and MIG, all of which were upregulated fivefold over those of controls (*p* < 0.001). In contrast, 15 of 68 (22.1%) cytokines were downregulated in HPS cases when compared to controls (Table 3), the greatest differences were observed for CXCL12, CCL21, CCL22, CCL27, and sCD40L (*p* < 0.001). Additionally, the majority of downregulated cytokines belonged to the homeostatic and inflammatory chemokine family. Of the 68 cytokines investigated, 28 (41.2%) were not statistically different when comparing cases and controls (Table 4).

**Table 2:** **Cytokines upregulated in HPS cases compared to healthy controls**.

Analyte	Case (pg/mL), *n* = 12	Control (pg/mL), *n* = 41	*p* Value
Upregulated In HPS serum			
IL-1α	537.7 ± 95.0	179.12 ± 15.7	0.0001
IL-2RA	455.3 ± 84.6	177.3 ± 7.3	0.0001
IL-2	11.7 ± 3.6	4.7 ± 0.8	0.005
IL-3	415.1 ± 86.4	140.5 ± 11.4	0.0001
IL-6	87.9 ± 22.7	10.8 ± 2.0	0.0001
IL-10	49.2 ± 31.5	15.7 ± 1.1	0.05
IL-12(p40)	927.3 ± 175.1	280.7 ± 22.9	0.0001
IL-17A*	23.3 ± 6.8	7.5 ± 0.2	0.0001
IL-17F*	74.3 ± 19.3	17.9 ± 4.8	0.0001
IL-18*	1651.6 ± 495.1	803.6 ± 66.7	0.006
IL-22*	42.1 ± 12.1	22.7 ± 0.5	0.004
CCL23*	705.9 ± 102.5	375.7 ± 37.6	0.0004
CXCL10	2834.2 ± 913.5	197.8 ± 18.8	0.0001
CX3CL1*	1456.6 ± 321.2	241.3 ± 13.2	0.0001
GM-CSF	55.3 ± 9.7	14.2 ± 2.5	0.0001
M-CSF	4811.7 ± 167.7	415.1 ± 26.5	0.0001
VEGF	179.2 ± 122.7	48.8 ± 6.1	0.05
MIF*	4779.9 ± 2229	540.6 ± 70.5	0.001
CXCL9*	2702.7 ± 891	355.0 ± 93.0	0.0001
TNFβ	227.9 ± 26	147.9 ± 12.9	0.007
IFNα	191.9 ± 26.2	123.6 ± 9.8	0.005
LIF*	346.7 ± 40.9	216.9 ± 10.7	0.0001
b-NGF*	122.0 ± 13.8	98.3 ± 3.9	0.03
SCF*	1180.8 ± 233.9	469.3 ± 30.9	0.0001
TRAIL*	391.9 ± 82.4	266.7 ± 14.8	0.02

**Table 3:** **Cytokines downregulated in HPS cases compared to healthy controls**.

Analyte	HPS (pg/mL), *n* = 12	Control (pg/mL), *n* = 41	*p* Value
Downregulated in HPS serum			
CCL1*	41.7 ± 0.3	43.3 ± 0.4	0.03
CCL5	1210.5 ± 230	5520.3 ± 670	0.001
CCL11	18.5 ± 0.9	47.1 ± 2.6	0.0001
CCL13*	37.1 ± 10.2	135.0 ± 14.1	0.0005
CCL17*	70.4 ± 31.5	241.4 ± 22.3	0.0004
CCL19*	156.2 ± 58.9	418.5 ± 38.1	0.001
CCL21*	979 ± 193	3504.6 ± 119	0.0001
CCL22*	276.2 ± 101	1112.8 ± 60.4	0.0001
CCL24*	356.7 ± 93.8	597.8 ± 49.5	0.02
CCL26*	16.4 ± 2.5	27.6 ± 1.9	0.005
CCL27*	319.8 ± 65.7	1411.4 ± 79.9	0.0001
CXCL6*	25.7 ± 44	48.2 ± 2.3	0.0002
CXCL12*	166.7 ± 32.7	2367.3 ± 104.3	0.0001
CXCL16*	183.4 ± 44.0	618.3 ± 27.9	0.0001
sCD40L	89.3 ± 54.4	2014.2 ± 128	0.0001

**Table 4:** **No significant difference in cytokine expression between HPS and healthy controls**.

Analyte	HPS, *N* = 12 (pg/mL)	Healthy control, *n* = 41 (pg/mL)	*p* Value
IL-1	4.59 ± 0.1	4.8 ± 0.2	0.52
IL-1RA	93.7 ± 50.7	50.4 ± 7.4	0.15
IL-1β	2.7 ± 0.1	7.9 ± 1.9	0.1
IL-4	64.9 ± 5.2	78.4 ± 5.2	0.18
IL-5*	6.3 ± 0.6	5.9 ± 0.1	0.27
IL-7	5.3 ± 2.1	5.5 ± 0.5	0.9
IL-9*	9.9 ± 1.7	19.7 ± 11.9	0.66
IL-13	8.7 ± 0.5	8.9 ± 0.34	0.59
IL-15	9.7 ± 4.1	5.7 ± 0.07	0.07
L-16*	252.2 ± 53.4	317.2 ± 40.9	0.4
IL-21	30.7 ± 8.7	30.1 ± 6.0	0.96
IL-23	102.6 ± 25.3	95.1 ± 17.4	0.83
IL-25*	1.6 ± 0.3	2.4 ± 0.3	0.2
IL-31*	18.5 ± 3.2	21.4 ± 2.2	0.49
IL-33*	402.6 ± 125.7	723.3 ± 100.0	0.11
CCL3	18.3 ± 3.7	43.9 ± 9.5	0.15
CCL7*	196.9 ± 27	169.1 ± 23.1	0.55
CCL8	75.8 ± 11.3	95.8 ± 6.8	0.15
CXCL1*	215.9 ± 34.9	232.4 ± 13.4	0.6
CXCL2*	236.8 ± 37.7	302.1 ± 25.3	0.2
CXCL5*	1085.8 ± 230.1	798.1 ± 90.6	0.43
CXCL11*	23.5 ± 5.2	41.3 ± 10	0.35
FGF*	14.8 ± 1.3	20.7 ± 2.7	0.24
GCSF	26.2 ± 13	26.4 ± 3.3	0.98
HGF*	973.7 ± 284.6	869.9 ± 82.4	0.64
IFNγ	20.1 ± 5.0	15.4 ± 1.6	0.24
DCGF-β*	6605.8 ± 1808	4692.7 ± 353.1	0.1
PDGF	889.9 ± 302	1095.5 ± 62.1	0.29

### Analysis of serum cytokines in early vs. late stage HPS

In order to investigate the possibility that differential expression of cytokines occurs between subjects with early and late stage HPS, we compared these two subgroups with each other and to healthy controls. Surprisingly, we observed only five cytokines to be differentially expressed between the two subgroups of HPS cases (Table 5). Of these, median IL-33 and CXCL6 levels were greater in the early stage subjects whereas median CCL23, CXCL1, and TNF-β were greater in the late stage subjects. As expected, differences in cytokine expression between subgroups and controls were consistent with differences observed between total HPS cases and controls (data not shown).

**Table 5:** **Serum cytokine profile during early and late stages of HPS**.

Analyte	HPS early (pg/mL)	HPS late (pg/nL)	Control (pg/mL)	*p* Value*	*p* Value**	*p* Value***
IL-1α	316.3 ± 60.4	545 ± 98.1	179.12 ± 15.7	0.006	0.0001	
IL-2RA	345.5 ± 92.5	454.9 ± 44.2	177.3 ± 7.3	0.001	0.0001	
IL-2	11.9 ± 5.5	10.1 ± 4.6	4.7 ± 0.8		0.02	
IL-3	269.3 ± 39.8	331.6 ± 87.2	140.5 ± 11.4	0.0004	0.0002	
IL-6	50.4 ± 30.5	109.9 ± 25.9	10.8 ± 2.0	0.003	0.0001	
IL-10	34.6 ± 12.4	14.6 ± 0.5	15.7 ± 1.1		0.02	
IL-12(p40)	613.9 ± 77.3	815.1 ± 196.3	280.7 ± 22.9	0.0001	0.0001	
IL-15	13.8 ± 7.5	5.6 ± 0.4	5.7 ± 4.1	0.008		
IL-17A	20.1 ± 6.2	24.6 ± 10.5	7.5 ± 0.2	0.0001	0.001	
IL-17F	61.0 ± 24.8	52.7 ± 10.7	17.9 ± 4.8	0.009	0.03	
IL-22	32.2 ± 12.5	55.8 ± 24.4	22.7 ± 0.5	0.0005		
IL-33	651.5 ± 179.2	74.5 ± 19.3	723.3 ± 100			0.03
CCL5	1339.8 ± 409.3	1168.7 ± 182.5	5520.3 ± 670	0.02	0.05	
CCL11	20.0 ± 1.3	17.5 ± 0.6	47.1 ± 2.6	0.0002	0.0009	
CCL17	124.1 ± 51.5	17.7 ± 5.6	241.4 ± 22.3		0.003	
CCL19	250.1 ± 64.4	126.3 ± 32.9	319.6 ± 38.1		0.02	
CCL21	732.9 ± 136.8	1185.5 ± 303.1	3504.6 ± 119	2.8E-11	0.0001	
CCL22	458.6 ± 159.4	108.5 ± 39.9	1112.8 ± 60.4	0.0004	0.0001	
CCL23	489.8 ± 112.4	990.7 ± 122.7	375.7 ± 37.6		0.0001	0.02
CCL24	229.5 ± 53.3	608.2 ± 180.2	597.8 ± 49.5		0.007	
CCL26	18.5 ± 4.3	13.9 ± 2.6	27.6 ± 1.9		0.03	
CCL27	306.4 ± 91.9	391.2 ± 97.1	1411.4 ± 79.9	0.0001	0.0003	
CXCL1	470.0 ± 50.8	954.3 ± 176.8	232.4 ± 13.4		0.03	0.01
CXCL5	2005.6 ± 1072.8	225.0 ± 49.9	708.1 ± 90.6	0.02		
CXCL6	32.9 ± 8.3	21.3 ± 10.9	48.2 ± 2.3		0.04	0.003
CXCL10	2785.2 ± 146.2	3843.1 ± 1266	197.8 ± 18.8	0.0001	0.0001	
CXCL12	191.1 ± 47.2	181.0 ± 42.6	2367.3 ± 104.3	0.0001	0.0001	
CXCL16	201.5 ± 73.8	175.2 ± 29.9	618.1 ± 27.9	0.0001	0.0001	
CX3CL1	1020.4 ± 440.6	1710.3 ± 309.1	241.3 ± 13.2	0.0001	0.0001	
GM-CSF	67.3 ± 9.7	43.6 ± 16.6	14.2 ± 2.5	0.0001	0.005	
DCGF-β	4611 ± 1036.1	11215.6 ± 3269	4692.7 ± 353.1		0.0002	
LIF	253.3 ± 39.1	386.8 ± 50.4	216.9 ± 10.7		0.0001	
M-CSF	1721.7 ± 475.2	3563.8 ± 1221.1	415.1 ± 26.5	0.0001	0.0001	
MIG	2924.6 ± 1596.1	3152.6 ± 2746.3	355.0 ± 93.0	0.0003	0.0001	
MIF	1977.3 ± 540.6	666.2 ± 200.9	540.6 ± 70.5	0.008	0.0001	
sCD40L	157.8 ± 85.1	15.7 ± 4.3	2014.2 ± 128	0.0001	0.0001	
SCF	798.8 ± 207.6	1390.8 ± 443.1	469.3 ± 30.9	0.006	0.0001	
TNFβ	167 ± 16.4	226.9 ± 16.1	147.9 ± 12.9			0.04
VEGF	286.5 ± 48.8	101.4 ± 28.5	48.86.1	0.01	0.02	

*p value early phase to control; **p value late phase to control; ***p value early to late phase.

### Classification of cytokines by importance

Given the complex interactions of cytokines with immune and non-immune cells, clarification of how distinct cytokines contribute to a pathological situation is often difficult to resolve. In order to provide insight into this issue, we implemented the machine logic algorithm RF to analyze our data set and potentially identify the most important cytokines that define this disease. For our analysis, 500 random decision trees were constructed with six predictors at each node, and auto-bootstrap out-of-bag sampling was implemented to test the accuracy of model. This model accurately identified HPS cases with 100% specificity and 73.81% sensitivity (Table 6). The 10 most significant cytokines for delineating HPS in decreasing order of importance are: M-CSF, CXCL16, sCD40, CXCL12, CCL22, IL-1a, CCL21, IL-12p40, CCL17, and IL-1b.

**Table 6:** **Random forest analysis of serum cytokines in HPS vs. controls**.

Variable	Score (%)	Changes in HPS serum	Variable	Score (%)	Changes in HPS serum
M-CSF	100.0000	Upregulated	IL-17F	19.4733	Upregulated
CXCL16	98.7888	Downregulated	CCL3	19.1369	Unchanged
sCD40L	96.8968	Downregulated	CCL1	18.8358	Downregulated
CXCL12	85.5322	Downregulated	CXCL11	18.5085	Unchanged
CCL22	78.4301	Downregulated	DCGFB	17.9953	Unchanged
IL-1α	74.0061	Upregulated	IL-4	17.7238	Unchanged
CCL21	70.3732	Downregulated	IL-25	17.4077	Unchanged
IL-12(p40)	62.9938	Upregulated	IL-33	16.6915	Unchanged
CCL17	62.8689	Downregulated	CCL7	16.5016	Unchanged
IL-1β	61.4314	Unchanged	TRAIL	15.7741	Upregulated
CCL5	61.0088	Downregulated	IL-9	14.3617	Unchanged
IL-3	58.5351	Upregulated	IL-18	12.0276	Upregulated
CCL13	58.0210	Downregulated	IL-7	10.5668	Unchanged
CXCL9	52.4830	Upregulated	IL-22	10.2808	Upregulated
CXCL10	50.3664	Upregulated	CXCL2	9.3174	Unchanged
CCL11	48.5759	Downregulated	MIF	8.8718	Upregulated
CCL27	46.5450	Downregulated	IL-16	8.8434	Unchanged
CXCL5	46.0115	Unchanged	b-NGF	7.9004	Upregulated
CX3CL1	45.6097	Upregulated	IL-31	7.0565	Unchanged
GM-CSF	43.1944	Upregulated	IL-10	6.3552	Upregulated
IFNα	41.2777	Upregulated	CCL8	6.1227	Unchanged
LIF	40.7034	Upregulated	IL-17A	5.3501	Upregulated
CCL24	39.4832	Downregulated	INFG	4.9877	Unchanged
IL-2RA	38.9993	Upregulated	GCSF	4.4335	Upregulated
PDGF	36.2886	Unchanged	IL-1	4.1642	Unchanged
CCL19	34.6337	Downregulated	FGF	4.0244	Unchanged
IL-6	31.5406	Upregulated	HGF	3.9312	Unchanged
CXCL6	30.4604	Downregulated	IL-1RA	3.5669	Unchanged
IL-15	25.5225	Unchanged	CXCL1	3.3770	Unchanged
TNFβ	24.6500	Upregulated	IL-5	2.6487	Unchanged
SCF	24.2768	Upregulated	IL-23	2.2930	Unchanged
IL-2	22.9123	Upregulated	VEGF	0.9825	Upregulated
CCL26	21.2675	Downregulated	IL-13	0.0038	Unchanged
CCL23	20.7866	Upregulated			

## Discussion

The microvascular endothelium is principal target of hantavirus infection in humans and its infection in lung tissue results in significant pathology (26). Infection of endothelial cells leads to increased vascular permeability without an observable cytopathic effect; therefore, the pathogenesis of HPS is likely indirect with immune responses, such as cytokine production, playing an important role. The cytokines that we observed to be upregulated in the serum of HPS cases are involved in a number of antiviral defense mechanisms including proliferation, maturation, and activation of leukocytes, as well as survival of leukocytes, and regulation of endothelial monolayer permeability (Table 2). High levels of IL-1α, IL-6, MIF, and TNF-β suggest a strong proinflammatory milieu in the serum of HPS cases, thus promoting both inflammation and activation of immune responses. We also observed stem cell proliferation factors to be upregulated, potentially promoting the proliferation and differentiation of subsets of immune effector cells. For example, proliferation of myeloid progenitors is strongly supported by IL-3, GM-CSF, and M-CSF. Increased serum concentrations of GM-CSF and M-CSF also suggest proliferation of monocytes and granulocytes (neutrophils, eosinophils, and basophils). Upregulation of the pluripotent factor, SCF, was also observed in association with HPS, suggesting increased proliferation of T lymphocytes, NK cells, and dendritic cells.

We observed a subset of 15 serum cytokines to be downregulated in our HPS cases (Table 4). Twelve of these cytokines are involved in chemotaxis of lymphocytes, such as B cells, T cells, and NK cells, to sites of infection. Some of these cytokines, including CCL22, CXCL12, and CCL17, are associated with activation of Th2-type immunity and are potent recruiters of Th2 cells to the lungs, as well as activators of pre-B cells (27–29). A number of cytokines identified as differentially expressed in the present study are consistent with putative immune responses of lung tissue. For example, we observed the upregulation of serum IL-17F, CXCL16, and IL-22, which are involved in the regulation of leukocyte migration into lung tissue, as well as lung tissue repair (30–33). Upregulation of IL-17F has also been observed in the lung tissue of asthmatic cases and its level positively correlated with disease severity (30, 34, 35). Overexpression of IL-17F promotes neutrophil infiltration and increased airways sensitivity and thus has a significant impact on lung function (35). IL-22 is considered a key cytokine for mucosal tissue repair (36) and by activating antimicrobial responses in lung epithelial cells; it has been shown to be critical for host defense as well. Also, IL-22 promotes lung epithelial cell proliferation (37) and therefore, based on our analyses, the cytokine profile observed in our HPS cases is consistent with a pulmonary antimicrobial response and subsequent mononuclear cell migration into the lung.

The serum cytokine profile observed in our HPS subjects also suggests a mobilization of mononuclear immune effector cells (Table 2). IL-12(p40) is an autocrine chemoattractant released by activated macrophages and promotes Th1-type immunity (38, 39). Additionally, serum levels for several potent T lymphocyte and NK chemoattractants were upregulated, including CXCL10, MIG, and CCL23 (15, 16, 40, 41). MIF and VEGF, which are regulators of mononuclear cell transendothelial migration, were upregulated as well. Migration of leukocytes can also be facilitated by the upregulation of adhesion molecules on the surface of endothelial cells in response to VEGF, IL-1α, and IL-6 (42, 43). MIF and VEGF promote expression of the adhesion molecules, E-selectin, ICAM-1, and VCAM-1, and increase vascular permeability (44, 45). Additionally, VEGF can decrease tight junctions between endothelial cells enabling transmigration of immune effector cells (42, 46). The observed increased serum levels of CXCL1, which may lead to release of VEGF-A from hantavirus-activated endothelial cells, further suggests that upregulation of VEGF plays a role in HPS (47, 48).

Cytokines including CXCL10, MIF, MIG, IL-12(p40), IL-17A, and CCL23 are known to promote proliferation and migration of mononuclear immune cells, such as T lymphocytes, NK cells, monocytes, and dendritic cells (15, 49–51). Consequently, our data support the previous observations of others whereby mononuclear cell and immunoblasts are the principal cellular infiltrate in the lungs of HPS cases (12). Nevertheless, the observed cytokine expression also is consistent with the activation and migration of neutrophils. Previous studies suggest that the cytokines, IL-17F, VEGF, CXCL1, GM-CSF, and IL-22, promote neutrophil migration and lung tissue repair (52–54). These data corroborate a previous report by Mori et al., who observed low-level neutrophil infiltration in the lungs of HPS case (12). Interestingly, serum level of CXCL8, the prototype neutrophil chemoattractant, was not significantly elevated in the HPS cases in our study; however, it was identified as one of the top 10 cytokines by our RF analysis, suggesting its expression, or lack thereof, plays an important role in HPS pathology. Our data further suggest that a Th17 shift occurs in HPS (55). In the presence of IL-23, non-Th17 cells can produce IL-17 (56); however, we observed no differential expression of serum IL-23 in HPS cases. Therefore, it is likely that activated Th17 lymphocytes were the source of IL-17 in the serum of our HPS cases.

Expression of IL-17 and IL-22 in HPS suggests a developing antimicrobial state in the lung. It has been reported that IL-17 and IL-22 activate β-defensins and the S100 family of proteins (52, 57). *In vivo* studies using knockout mice have demonstrated that IL-17 and IL-22 are crucial for bacterial defense in the lung (58, 59). Furthermore, it has been reported that IL-17R signaling is mandatory for the establishment of an antibacterial response to *M. pneumoniae*, systemic fungal infection, *B. fragilis*, and *E. coli* (60–63). Consistent with this statement, a protective role for IL-22 was recently reported for experimental influenza A virus infection (64).

We also observed a subset of cytokines involved in the regulation of platelet counts and function to be downregulated in the serum of our HPS subjects, including sCD40L, CCL5, CCL22, and CXCL12 (Table 3). Consistent with our observations and the pathophysiology of HPS, CXCL12 and CCL22 act on platelets to rapidly stimulate their adhesion (65), and CCL5 and sCD40L are released by activated platelets (66–68). Wenzel and coworkers reported that serum levels of sCD40L closely correlate with platelets counts and that they are increased upon thrombocyte transfusion (69). Viallard et al. also reported a correlation between thrombocyte counts and serum sCD40L, implying that it may be used as a surrogate marker for platelet counts (66). Decreased thrombocyte counts are also well documented in association with HPS (2, 70) and our observation of downregulated sCD40L presents a potential biomarker for the thrombocytopenia. Notwithstanding, decreased serum CCL22 might also reflect the development of the thrombocytopenia observed in HPS cases. It has been shown that CCL22 is capable of aggregating platelets in the presence of low concentrations of thrombin or adenosine diphosphate (ADP), and can rapidly stimulate platelets adhesion (65). It is noteworthy that endothelial cells do not produce this cytokine; dendritic cells are the main source of CCL22 (71). Therefore, the thrombocyte aggregation and depletion observed in HPS may be the result of cytokine-driven immune responses.

Serum levels of CCL21 and CCL27 were also downregulated in the serum of our HPS subjects. These cytokines have tissue-specific activity; for example, CCL21 orchestrates dendritic cell and T cell trafficking to the lymph nodes (72–74) and CCL27 regulates migration of immune effector cells to the skin (75). Taken together, these findings suggest that the cytokines expressed during HPS promote lung tissue infiltration while reducing leukocyte trafficking to other organs and tissues.

In order to investigate the contribution of each respective cytokine to the disease process, we conducted classification analysis by RF. Of the 10 most important cytokines identified by this analysis, 3 were significantly upregulated, as determined by Mann–Whitney analysis; however, we also observed 6 to be downregulated. This observation underscores the importance of cytokine inhibition in the disease process and further suggests that depressed serum cytokine expression may be an important biomarker for monitoring disease progression.

Overall, the majority of downregulated serum cytokines were associated with Th2-type immune activation; these included CCL21, CCL17, CCL13, and CCL11. Furthermore, the cytokines significantly upregulated in HPS cases were those promoting Th1-type immunity; these included CXCL9, CXCL10, and IL-12(p40). The cytokines M-CSF, CXCL12, IL-3, LIF, GM-CSF, CCL24, which facilitate activation, differentiation, and bone marrow mobilization of myeloid progenitors, were also identified by RF analysis to differentiate HPS cases from controls. RF analysis further identified chemokines associated with platelet aggregation as important in differentiating cases from controls. Interestingly, sCD40L and CXCL12 were ranked, respectively, as the third and fourth most import cytokine in our RF analysis. Chemokines, such as sCD40L, CXCL12, and CCL17, which are stored in platelet granules, are released upon platelet aggregation, a process that is critical in HPS pathology (2, 76–78). Accordingly, nadir platelet counts in HPS may explain low serum CXCL1, CCL17, and sCD40. Taken together, RF analysis supports the supposition that HPS pathogenesis may be characterized by Th1-type immune responses and thrombocytopenia.

In summary, our data suggest that HPS is characterized by a serum cytokine profile that is consistent with putative immune responses in lung tissue. Strong activation of mononuclear immune effectors including T lymphocytes, NK cells, and dendritic cells is also suggested by this cytokine profile. Additionally, our data imply that decreased counts and increased aggregation of thrombocytes in HPS might be explained in part by the immune response to viral infection. Lastly, to the best of our knowledge, our data provide the first evidence of Th17 lymphocyte activation in association with HPS. The data presented in this study are suggestive of putative *in vivo* immune mechanisms and may identify the role of these cytokines in HPS pathophysiology; however, future studies using animal models would be necessary to definitively confirm their involvement.

## Conflict of Interest Statement

The authors declare that the research was conducted in the absence of any commercial or financial relationships that could be construed as a potential conflict of interest.
